# Hybrid Deep Learning Approach for Automatic Detection in Musculoskeletal Radiographs

**DOI:** 10.3390/biology11050665

**Published:** 2022-04-26

**Authors:** Gurpreet Singh, Darpan Anand, Woong Cho, Gyanendra Prasad Joshi, Kwang Chul Son

**Affiliations:** 1 Department of Computer Science and Engineering, Chandigarh University, Mohali 140413, India; gps_ghotra@yahoo.com (G.S.); darpan.anand.agra@gmail.com (D.A.); 2 Department of Software Convergence, Daegu Catholic University, Gyeongsan 38430, Korea; wcho@cu.ac.kr; 3 Department of Computer Science and Engineering, Sejong University, Seoul 05006, Korea; 4 Department of Information Contents, Kwangwoon University, Seoul 01897, Korea

**Keywords:** deep learning, musculoskeletal abnormalities, prediction, convolutional neural network, machine learning, artificial intelligence, radiography images, compression, progressive resizing

## Abstract

**Simple Summary:**

Musculoskeletal disorder is affecting a large population globally and is becoming one of the foremost health concerns. The treatment is often expensive and may cause a severe problem in the case of misdiagnosis. Therefore, a reliable, fast, and inexpensive automatic recognition system is required that can detect and diagnose abnormalities from radiographs to support effective and efficient decision making for further treatment. In this work, the finger study type from the MURA dataset is taken into consideration owing to the fact the existing models were not able to give the desired performance and accuracy in detecting abnormalities in finger radiographs. Herein, a novel deep learning model is proposed, wherein after the preprocessing and augmentation of the finger images, they are fed into a model that learns the discriminative features through multiple hidden layers of dense neural networks and classifies them as normal or abnormal radiographs. The achieved result outperforms all existing state-of-the-art models, making it suitable for clinical settings. This will help society in the early detection of the disorder, which reduces the burden on radiologists and reduces its long-term impact on a large population.

**Abstract:**

The practice of Deep Convolution neural networks in the field of medicine has congregated immense success and significance in present situations. Previously, researchers have developed numerous models for detecting abnormalities in musculoskeletal radiographs of upper extremities, but did not succeed in achieving respectable accuracy in the case of finger radiographs. A novel deep neural network-based hybrid architecture named ComDNet-512 is proposed in this paper to efficiently detect the bone abnormalities in the musculoskeletal radiograph of a patient. ComDNet-512 comprises a three-phase pipeline structure: compression, training of the dense neural network, and progressive resizing. The ComDNet-512 hybrid model is trained with finger radiographs samples to make a binary prediction, i.e., normal or abnormal bones. The proposed model showed phenomenon outcomes when cross-validated on the testing samples of arthritis patients and gives many superior results when compared with state-of-the-art practices. The model is able to achieve an area under the ROC curve (AUC) equal to 0.894 (sensitivity = 0.941 and specificity = 0.847). The Precision, Recall, F1 Score, and Kappa values, recorded as 0.86, 0.94, 0.89, and 0.78, respectively, are better than any of the previous models’. With an increasing appearance of enormous cases of musculoskeletal conditions in people, deep learning-based computational solutions can play a big role in performing automated detections in the future.

## 1. Introduction

Good health is defined as a ‘State of complete physical, social and mental wellbeing and not merely the absence of disease, or infirmity’ [1], and a natural corollary of being healthy is the healthcare facilities available to a person for enjoying good health. Today, we have a vast quantity of unstructured data produced by health care systems and hospitals, such as medical imaging data, genomic information, and free text and data streams from monitoring devices [2]. Processing medical data manually is often time-consuming, and the chance of errors in interpretation is not irrelevant. For instance, it has been estimated that daily error rates and discrepancies in radiology are greater than 3–5% [3]. Moreover, radiologist workloads increase with more images, greater case volumes, increased complexity, and less time to work, possibly leading to radiologist burnout [4]. Therefore, it is the need of the hour that healthcare should explore advanced technologies to assist primary care physicians in improving patient care quality. The goal of healthcare is to become more personal, predictive, preventative, and participatory, and artificial intelligence (AI) can make major contributions in these directions. AI technologies can perform a wide array of functions, such as aiding in diagnosis generation and therapy selection, making risk predictions and stratifying disease, reducing medical errors, and improving productivity [5,6].

Back in the 1950s, the fathers of the field, Minsky and McCarthy, described artificial intelligence as any task performed by a machine that would have previously been considered to require human intelligence. Artificial intelligence (AI) has recently experienced an era of explosive growth across many industries, and healthcare is no exception [7]. Regardless of the specific technique, the general aim of these technologies in medicine is to use computer algorithms to learn features from a large volume of healthcare data [8] and then use it to assist clinical decision making [9]. It can also be equipped with learning and self-correcting abilities to improve its accuracy based on feedback. There are several ways in which AI-based technologies could be implemented into clinical practice. The first is as a screening tool or triage. For example, the radiology images could be analyzed by AI to find the probability of disease to decide which images should be interpreted first by the human radiologist [10], or it could determine which patients have vision-threatening conditions and require the urgent attention of an ophthalmologist [11] after examining retinal images. The increasing availability of healthcare data and rapid development of big data analytic methods have made possible the recent successful applications of AI in healthcare [12,13,14].

Musculoskeletal disorders are the foremost contributor to disability globally. Musculoskeletal diseases are not only becoming a growing burden for older people but are also prevalent across the life course, leading to radiologist burnout. An increased prevalence of musculoskeletal disorders can affect any part of the body, including the bones, muscles, joints, ligaments, etc. Growing primary care radiology facilities and radiologist workload make it significant to discover the usage of artificial intelligence to provide diagnostic support to increase the quality of patient care. The acquisition of information and actionable intuitions from complex, high-dimensional, and diverse radiographic data remains a vital contest in renovating health care. The clinical images to be examined hold a lot of evidence regarding the atomical structure to reveal effective diagnoses and aid specialists in selecting suitable treatments. Due to the rise in computing power and accessibility of enormous datasets in recent years, new machine learning algorithms are being produced that are able to match and even exceed humanoid performance in gradually complex tasks [15]. Inspired by human brains, deep learning algorithms are trained on data to learn discriminative features, which are now increasingly being used in radiological applications [16]. The modern improvements in deep learning technologies offer new effective standards for processing complex radiographic data by deploying more than one hidden/fully connected processing layer to train a model [17].

**Figure 1:**
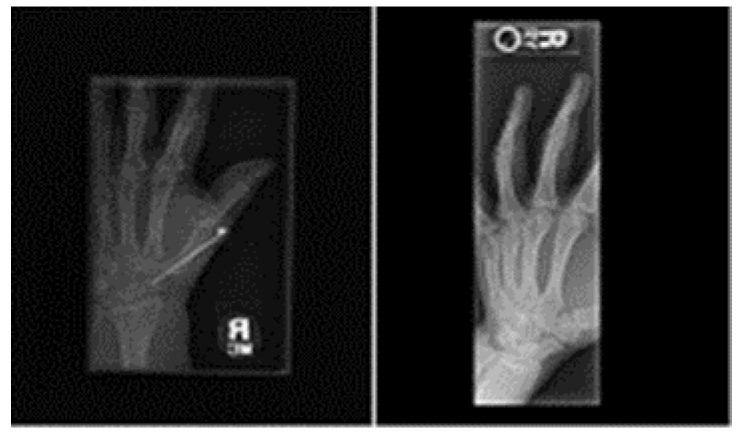
Normal and abnormal finger image [18].

The rationale is to build an efficient model for the prediction of Musculoskeletal Abnormalities using a radiographic image. This research work focuses primarily on finger radiographic images, i.e., a disorder in a finger. A normal radiograph with no disease and an abnormal radiograph with arthritis is shown in Figure 1. The model categorizes the medical images as normal or abnormal in three main stages. Initially, lossless compression is applied to input images to reduce their size while preserving all their characteristics, then a deep learning model is trained on compacted images of size *n* × *n* to classify them as normal or abnormal, and lastly, the progressive resizing concept is employed to improve the accuracy of the model by inserting an already-trained layer into a new dense network model that processes compressed images of size 2*n* × 2*n* to predict the final output.

## 2. Related Work

This section provides an overview of related work conducted by various researchers previously. Rajpurkar P. et al. [18] in their work used 169-layer CNN to detect abnormalities in upper extremities in musculoskeletal images, but the accuracy achieved by the model in the case of finger radiograph was only 38.9%. Chada G. [19] in his paper proposed a CNN model using deep transfer learning to obtain a better result than before. Verma M. et al. [20] worked on the automatic detection of abnormality in lower extremity radiographs using a densely connected CNN model. In [21], the author used VGG-19 and ResNet architecture to build a deep CNN model, which achieved an accuracy of 82.13%. In [22] the author implemented a deep learning-based model on ensembles of Efficient-Net architectures to automate the detection process. In [23], the author combined the GNG network and VGG model to classify and detect abnormalities in bone X-rays. In [24], an ensemble learning approach was used to build a deep CNN model for detecting abnormalities in upper extremities. In [25], deep transfer learning, along with some data preprocessing techniques, was used to build a CNN model for detecting abnormalities in upper extremities, but the model achieved the highest accuracy of 67.05% in the case of finger radiographs. In [26,27], the various models of machine learning based on deep learning were reviewed, and in [28,29] the deep learning approach was applied to COVID-19 detection. Table 1 summarizes the literature review performed.

Gap Analysis: After going through the various literature mentioned in the table, it was observed that (a) the best performance of the model proposed by Rapjpurkar et al. [18] is less than the worst performance of radiologists on different study types of the MURA dataset. (b) The model performance on finger study type is not giving promising results in detecting abnormalities. (c) Ensemble learning is used in the literature [21,24] and has significant overhead in terms of time consumption and computation time, which needs to be addressed. The general observation about the available models for the same cause is features that are used for training. There is a huge similarity between the true and false cases, which impacts the accuracy of the system. Therefore, in this proposed work, the progressive resizing concept is used to train our CNN model to automatically detect abnormalities in the finger radiograph, and our result outperformed all existing models. This proposed model uses CNN for automatic detection because the dataset is quite large and, while comparing the outputs with other conventional machine learning models, it will give better results. Moreover, the proposed model can be trained using multiple hidden layers to achieve a higher accuracy and then can be used in reality to reduce radiologist workload. Due to the heavy load on the radiologists and health executives, the primary screening can be performed through this model. On the basis of the severity, the case can be forwarded to an expert in the case of an emergency. This is the way to prioritize the cases to handle the conditions optimally to provide better health consultancy to all.

## 3. Materials and Methods

This section discusses the method and dataset used in the research.

### 3.1. Dataset

The Mura dataset [18] was used for the study. It is one of the largest openly accessible datasets for abnormality detection in upper extremity musculoskeletal radiographs. It holds 40,561 radiographs from 14,863 studies acquired from the Picture Archive and Communication of Stanford Hospital. The dataset contains seven standard upper limb study types: finger, hands, wrists, forearms, elbows, humerus, and shoulders. The studies of different patients are separated as positive studies and negative studies for each type. A sample set of finger radiographs only was taken from it. The radiographs of interest were divided into Training, validation, and test dataset modules. Table 2 shows the number of radiographs, i.e., normal and abnormal, for each set chosen for study. The model was initially trained on the training set and then validated on the images of the validation set. Finally, the model was evaluated on the test set to verify how well it is able to predict the abnormalities.

### 3.2. Experimental Setup

The work was implemented using Python (ver. 3.6.9, Python Software Foundation, Wilmington, DE, USA) [30]. Keras library (with TensorFlow backend) was used for our study as it contains tools and techniques enabling fast experimentation. It runs seamlessly on CPU and GPU. Sklearn provides a range of supervised and unsupervised learning algorithms, Pandas for data analysis and manipulation, Numpy for multidimensional arrays and metrics, and Matplot libraries comprehensive 2D/3D plotting were also used. Google Colab is a free cloud service that provides GPU and TPU to execute deep learning models used to implement the proposed model.

### 3.3. Convolutional Neural Network

**Figure 2:**
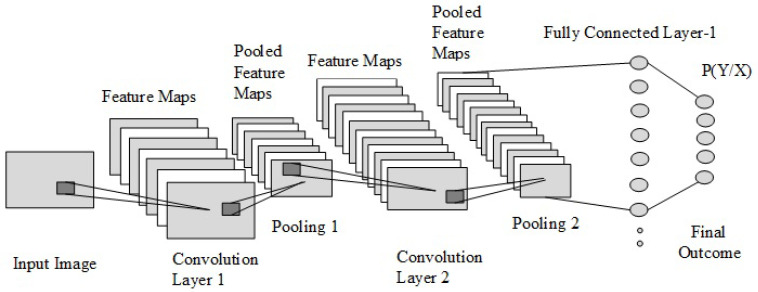
The structure of a convolutional neural network.

A convolutional neural network [31] is a widely used approach for image recognition and classification problems. The foundation of CNNs is their capability to operate with insignificant human engineering, aligning well with artificial intelligence. CNNs are analogous to regular neural networks [32,33]. They contain a number of neurons that have learnable weights and biases. Every neuron accepts some inputs, performs a dot product, and is followed by non-linearity (optionally). The structure of CNN is shown in Figure 2, which consists of an input layer followed by the convolution, pooling, fully connected, and output layers.

#### 3.3.1. Convolution Layer

**Figure 3:**
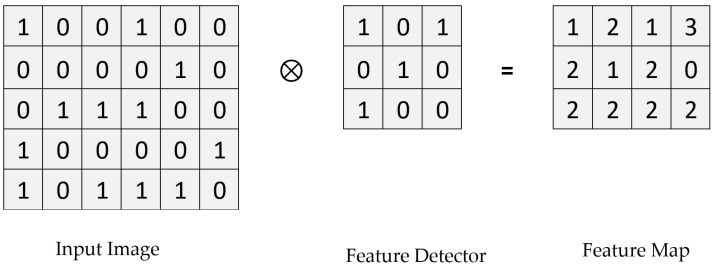
Convolution layer operation.

In this, the input image is represented in the form matrices of 1′s and 0′s, and then the feature detector (i.e., filter) is applied to it to obtain the feature map, as shown in Figure 3. In order to obtain multiple convolved images (feature maps), various kinds of feature detectors are applied to the input image to extract different features [34,35].

Mathematically, a convolutional is a function derived from two given functions by integration that expresses how one function transforms the shape of the other.

(1) $$\left(f\times g\right)t\;\underset{=}{\mathrm{def}}\int^{\infty }\left(x\right)g\left(t-x\right)\mathrm{dx}$$

#### 3.3.2. ReLU Layer

**Figure 4:**
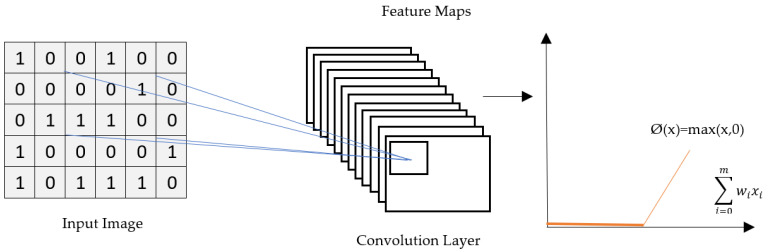
ReLU Layer Operation.

The rectified linear unit (ReLU) is an activation function that is ordinarily used in deep learning network models [36]. The purpose of applying a rectifier to a function is to increase the non-linearity in the image. If the function receives a negative value, it will return ‘0’, and for all of the positive values ‘z’, it returns that value. Figure 4 shows how the activation function is applied on feature maps. It helps a model account for interaction effects and non-linear effects.

(2) $$f\left(x\right)=\{\begin{array}{c} 0,x<0\\ 1,x\geq 0 \end{array}$$

#### 3.3.3. Pooling Layer

The objective of introducing a pooling layer in CNN is to attain spatial invariance by decreasing the resolution of the feature detectors [37]. Pooling operations such as sum pooling, average pooling, and mean pooling are executed autonomously on each feature map. It is important that it preserves the original feature and is still able to reduce the spatial size and number of parameters, and thus computation, in the network.

The subsampling function and max pooling function are shown in Equations (3) and (4), respectively.

(3) $$a_{j}=\tanh\left(\beta \underset{N\times N}{\sum }a^{n\times n}+b\right)$$

(4) $$a_{j}=max\left(a^{n\times n}\;u\left(n,\;n\right)\right)$$

The trainable scalar *β* is added to trainable bias *b* after multiplying it with the average over the input and passes the end result through non-linearity. The max function calculates the maximum in the neighborhood when a window function *u* (*n*, *n*) is applied.

**Figure 5:**
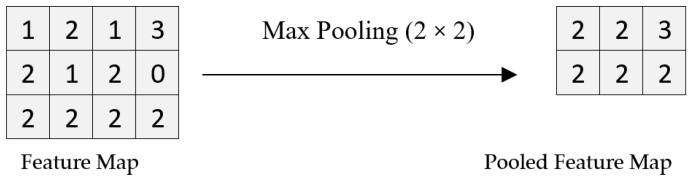
Max pooling.

A pooling operation is shown in Figure 5, in which a filter of 2 × 2 is applied to a feature map to obtain the pooled feature map.

#### 3.3.4. Flattening

**Figure 6:**
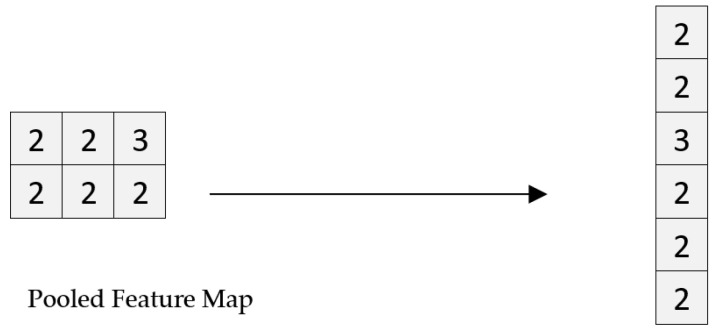
Flattening.

Flattening is translating the pooled data into a 1D array. The output of the convolution layers is flattened to generate a single feature vector [38]. It is then linked to the final classification model called a fully connected layer. The flattening operation is shown in Figure 6, where the pooled feature map is converted into a single vector.

#### 3.3.5. Fully Connected Layer

**Figure 7:**
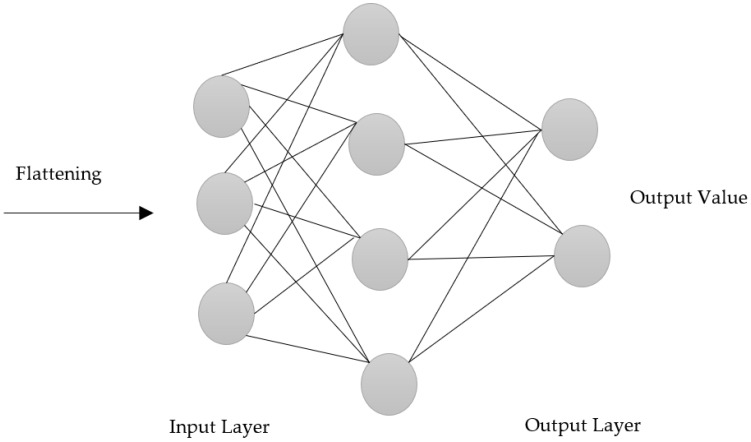
Fully connected layer.

In a convolution neural network, all hidden layers are fully connected with each other, i.e., each neuron at the first layer has a path to every other neuron to the next layer and so on [39]. The output of the convolution/pooling process is given to a fully connected layer, which is then used to categorize the radiographs into a tag. In order to define the most truthful weights, the fully linked part of CNN goes through its own backpropagation process. The weights received by each neuron prioritize their most-suitable tag. Lastly, the neurons ‘vote’ on each tag, and the classification decision depends on the winner of that vote. Figure 7 shows how each input node is connected to every node of the hidden layer, and every node of the hidden layer is further connected to every node in the output layer.

## 4. Proposed Model

In this section, implementation details of the proposed method are presented.

**Figure 8:**
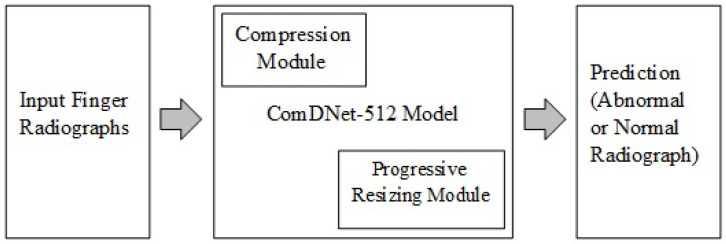
Abstract view of proposed ComDNet-512 model.

**Figure 9:**
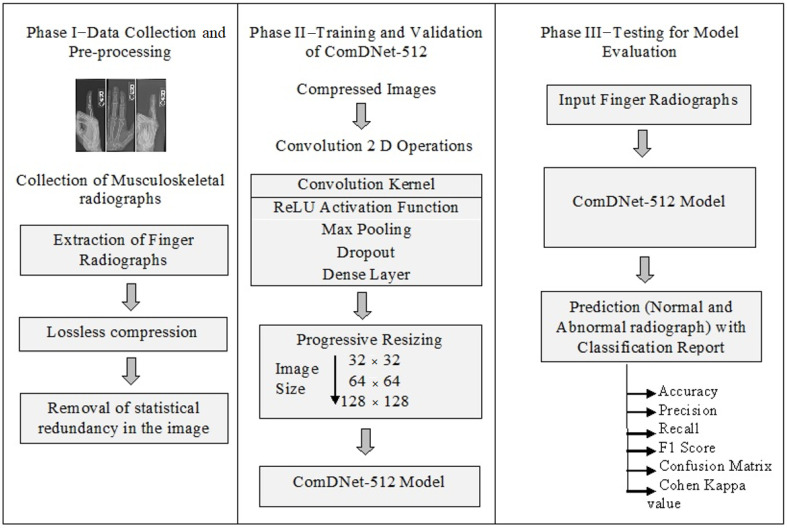
Detailed view of the proposed model.

A dense layer CNN in Python using Keras (with Tensor flow backend) was designed to detect an abnormality in the input finger image. The abstract and detailed view of the proposed model are shown in Figure 8 and Figure 9, respectively.

In phase I, data collection and preprocessing were performed. The finger X-ray radiographs were extracted from the MURA dataset [18]. Multiple processing functions such as rotation, shift, and flips were applied to train the deep learning model well to give better performance, which then went through compression to remove statistical redundancy. The lossless compression was applied to all the clinical images with no loss of fidelity of the original data. The statistical redundancy in the image was detached by applying compression [13]. The size of medical images is normally very large, which, if given as an input to a network for classification, would take a lot of time for training a model. Additionally, it allows the transmission of an image at very low bandwidths and minimizes the space requirements. Furthermore, the quality of images was not at all compromised as it would have resulted in very low model performance. In phase II, a dense layer network was built, and training and validation of the model are performed. Convolution 2D was the first layer of the proposed model and constructs a convolution kernel producing a tensor of outputs. The activation function ‘ReLU’ was applied to remove linearity. An Adam [35] is used as an optimizer for the study. An Adam optimizer involves a combination of two gradients’ decent methodologies to give better results in terms of optimization, while the general methods used for optimization include a single gradient descent method. Binary cross-entropy was used to compute training and validation loss. Max pooling with a filter of size 2 × 2 with a stride of 2 was used for downsampling the spatial dimensions of input. Dropout [34] equal to 0.2 was applied to the network to prevent over-fitting. Some specified percentage of neurons along with their incoming and outgoing edges were removed from the network. The concept of progressive resizing was then applied in order to improve the model accuracy. The model was initially trained on the image of size 32 × 32, then its trained layers were used to train the 64 × 64 model, and, lastly, the recently trained model was applied to the upscaled model of image size 128 × 128. There was an increase in the accuracy when moving from a small size image model to a large size model. In phase III, the model was tested against the testing set, and its classification report was generated to verify its performance.

## 5. Experiment

**Figure 10:**
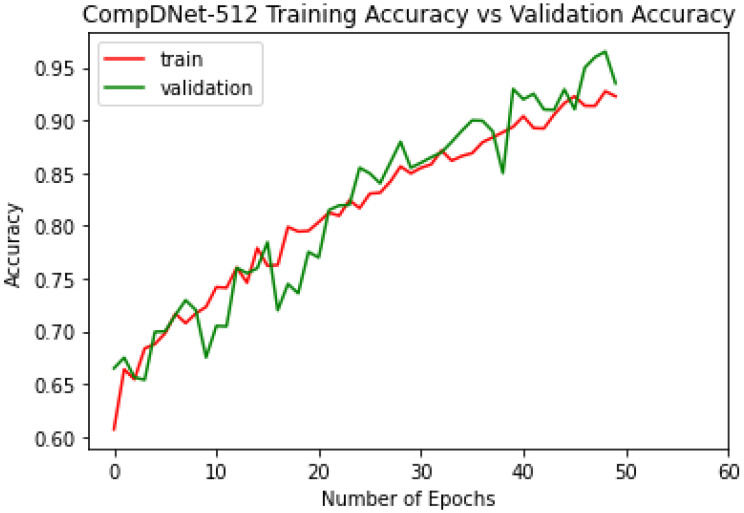
ComDNet-512 training and validation accuracy.

**Figure 11:**
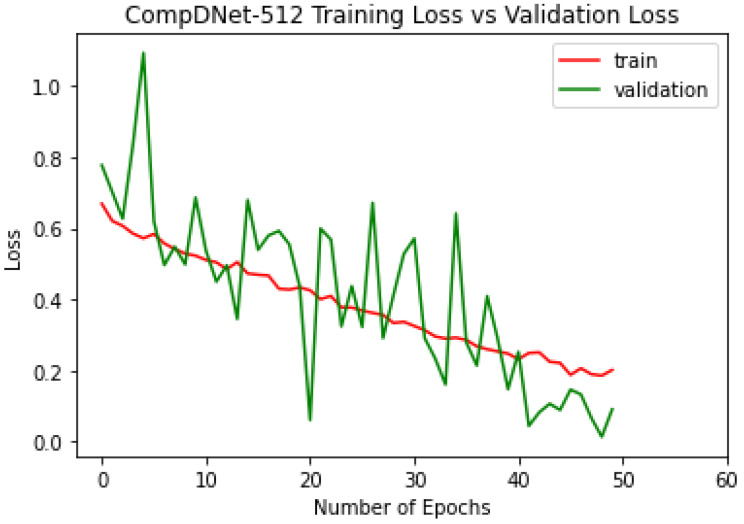
ComDNet-512 training and validation loss.

Training and Validation:

The ComDNet-512 model was trained on a total of 1702 normal and abnormal images and validated on 300 radiographs. The model was able to attain a validation accuracy of 93.51% with a validation loss of 0.09 and a training accuracy of 92.28% with a training loss of 0.20 over 50 epochs, which can be clearly seen in the Figure 10 and Figure 11.

The training process started in the proposed model on small-size images first, as they generalize well to the larger input size. A small image model is much faster to train, which saves more time that can be spent understanding and visualizing our dataset to remove irrelevant or ambiguous data. The hands-on result is that a model trained on a small image will learn fewer features, while it will learn more features on large images. The model learns more features when expanded from a small image to a large image, and its accuracy is also increased. The training and validation accuracy achieved by the model is shown below for different size images, which is shown in Table 3. It can clearly be seen that the accuracy increases as the size of the images increases, which validates the progressive resizing concept.

## 6. Results and Discussion

The ComDNet-512 model, after being trained and validated for 50 epochs on a small dataset of 2002, was then tested on the test dataset, which contains 170 images, i.e., a mixture of normal and abnormal finger radiographs, to verify its prediction accuracy. The model’s performance summary is shown in Table 4 below.

The performance of a classification model on a dataset for which correct values are well-known can be expressed in terms of the confusion matrix. It permits the visualization of the performance of an algorithm. Here in this study, the proposed model is a binary classifier, i.e., Class 1: Normal and Class 2: Abnormal. The test dataset contained 85 normal and 85 abnormal images. The following parameters were used to calculate the confusion matrix.
True Positive (TP):The original image was abnormal, and the model prediction was also abnormal.False Negative (FN):The original image was abnormal, but the model predicted it as normal.True Negative (TN):The original image was normal, and the model prediction was also normal.False Positive (FP):The original image was normal, but the model predicted it as abnormal.

**Figure 12:**
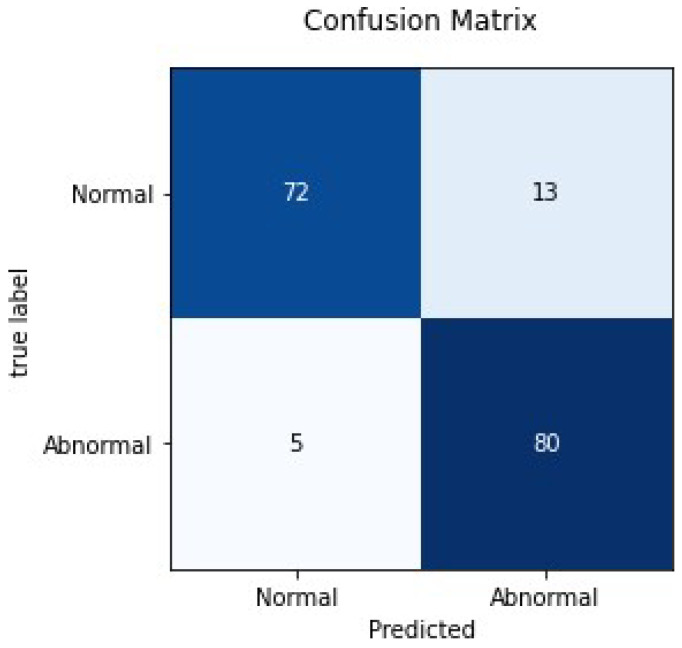
Confusion matrix for ComDNet-512.

**Figure 13:**
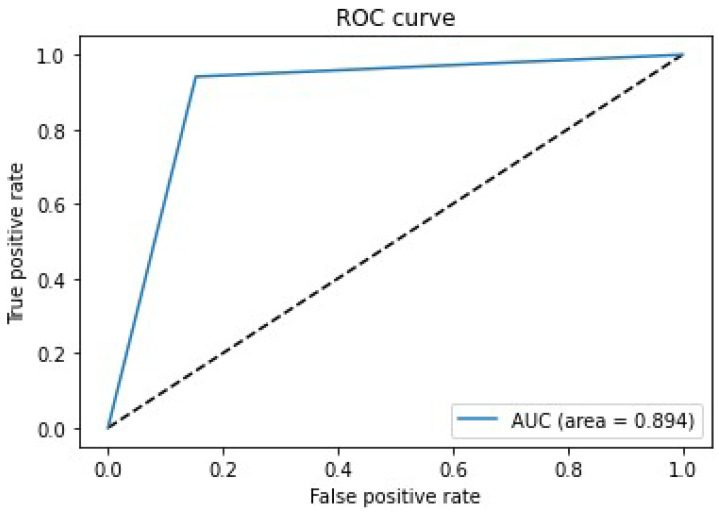
Receiver Operating Characteristics (ROC) Curve.

Figure 12 shows the confusion matrix for the proposed model when tested on a small test set of 170 images. It clearly shows that it correctly predicted abnormalities in 80 radiographs out of 85 images. Figure 13 shows the Receiver Operating characteristics (ROC) curve for a model when tested on the test dataset. It takes a true positive rate on the *x*-axis and a false positive rate on the *y*-axis. The farther the curve is from the diagonal, the more clearly it can differentiate between two classes. The model was able to achieve an area under the ROC curve (AUC) equal to 0.894. The false-negative rate (FNR) was 0.058, and false-positive rate (FPR) was 0.152, and overall sensitivity and specificity achieved by the model were 0.941 and 0.847, respectively.

Finally, the outcomes achieved by the proposed model were matched with the results of previously existing model detection of abnormalities on finger radiographs. This comparative analysis is shown in Table 5. As per the statistics shown in Table 5, the accuracy of models DenseNet-169, DenseNet-201, and InceptionResNetV2 were 75.70%, 76.57%, and 77.66%, respectively, whereas the proposed model gave an accuracy of 89.41%. It can be seen that the proposed ComDNet-512 model outperformed the existing model in every aspect.

The outcome of the proposed model was better than the existing state-of-the-art models in detecting the abnormality in the finger study type of MURA dataset. Further, the model can be trained for other study types in the future, taking segmentation and other machine learning approaches into consideration for feature extraction, and then performance can be compared with radiologist performance on all study types of the MURA dataset.

## 7. Conclusions and Future Scope

The ComDNet-512 model, employing the Deflate compression technique—dense learning with progressive resizing to optimize training on limited data, identified abnormalities in finger radiographs with an accuracy of 89.41%. When applied to finger radiographs, the three models outperformed already-existing models. The model was able to achieve an area under the ROC curve (AUC) equal to 0.894. The Precision, Recall, F1 Score, and Kappa values recorded as 0.86, 0.94, 0.89, and 0.78, respectively, are better than those any of the previous models. This model can be further retrained periodically on new incoming data to improve accuracy and hence can be used by a radiologist to perform automated detection. The research was carried out on a finger study type only, as existing models are not able to achieve a good result on finger radiographs. In the future, a bigger data size will be taken, along with the implementation of Hadoop for processing, and the model will be trained to detect abnormalities in all the study types included in the dataset. Further existing models were not implemented for all study types of the dataset, so in the future, a single model can be developed that can detect abnormalities in all the study types. There are various other optimizers and feature extraction methods available, and further work will include an analysis of segmentation and optimization techniques on the MURA dataset.

## Figures and Tables

**Table 1:** Related work.

Target Disease	Description	Technique Used	Findings	Reference
Abnormality Detection in upper extremities in musculoskeletal radiographs	DenseNet-169 Baseline models were used to detect and localize abnormalities.	169-layer CNN	The accuracy achieved by the model in the case of finger radiographs was 38.9%	[18]
Abnormality detection in humerus and finger radiograph.	DenseNet-169, DenseNet-201, and InceptionResNetV2 were implemented and evaluated on humerus and finger radiographs.	Deep Transfer Learning	The best accuracy achieved was 77.66% in finger radiographs.	[19]
Musculoskeletal disorder	Abnormality detection in lower extremity radiographs.	DenseNet-161	With an AUROC of 0.88, it can be utilized to identify diverse abnormalities in lower extremity radiographs.	[20]
Abnormality detection in upper extremities in a musculoskeletal radiograph	They used VGG-19 ResNet architecture to build a model for four types of study (elbow, wrist, finger, and humerus).	Deep CNN	The highest accuracy achieved by the model was 82.13%.	[21]
Abnormality detection in upper extremities in a musculoskeletal radiograph	Use of deep learning model based on ensembles of Efficient-Net architecture to automate the detecting process.	Deep Transfer Learning of ImageNet.	The accuracy achieved by EfficientNet-B3 for finger radiograph was 85.5%.	[22]
Abnormality detection	Two-stage method for bone X-ray classification and abnormality detection.	Combining GNG Network and VGG model.	The highest accuracy achieved by the model was 78.51%.	[23]
Abnormality detection in upper extremities in a musculoskeletal radiograph	A new calibrated ensemble approach based on three deep neural networks for detecting musculoskeletal abnormalities.	Ensemble Learning approach (ConvNet, ResNet, and DenseNet)	The highest accuracy achieved by the model was 83%.	[24]
Abnormality detection in upper extremities in a musculoskeletal radiograph	They applied data augmentation resizing and cropping for data preprocessing and used an updated version of the pre-trained model DenseNet-169 for abnormality detection.	Deep Transfer Learning	The highest accuracy achieved by the model was 67.05%.	[25]

**Table 2:** Training, validation and test datasets under study.

Training Set		Validation Set		Test Set	
Normal	Abnormal	Normal	Abnormal	Normal	Abnormal
3000	3000	1000	1000	85	85

**Table 3:** Training and validation accuracy and loss.

Filter Size	Training Accuracy	Loss	Validation Accuracy	Loss
32 × 32	84.32	0.33	86.45	0.32
64 × 64	88.92	0.25	92.51	0.25
128 × 128	92.28	0.20	93.51	0.09

**Table 4:** ComDNet-512 performance summary.

Accuracy	Precision	Recall	F1 Score	Kappa Value
89.41	0.82	0.97	0.89	0.74

**Table 5:** Comparative performance analysis of ComDNet-512 with state-of-the-art techniques.

Model	Accuracy (%)	Recall	Precision	F1 Score	Kappa
DenseNet-169 [19]	75.70	0.63	0.88	0.74	0.522
DenseNet-201 [19]	76.57	0.69	0.84	0.76	0.535
InceptionResNetV2 [19]	77.66	0.72	0.84	0.78	0.555
ComDNet-512	89.41	0.94	0.86	0.89	0.788

## Data Availability

Not applicable.

## References

[1] Callahan D. (1973). The WHO definition of health. Hastings Cent. Stud..

[2] Senders J.T., Arnaout O., Karhade A.V., Dasenbrock H.H., Gormley W.B., Broekman M.L., Smith T.R. (2018). Natural and artificial intelligence in neurosurgery: A systematic review. Neurosurgery.

[3] Brady A.P. (2017). Error and discrepancy in radiology: Inevitable or avoidable?. Insights Imaging.

[4] Harolds J.A., Parikh J.R., Bluth E.I., Dutton S.C., Recht M.P. (2016). Burnout of radiologists: Frequency, risk factors, and remedies: A report of the ACR Commission on Human Resources. J. Am. Coll. Radiol..

[5] Jiang F., Jiang Y., Zhi H., Dong Y., Li H., Ma S., Wang Y., Dong Q., Shen H., Wang Y. (2017). Artificial intelligence in healthcare: Past, present and future. Stroke Vasc. Neurol..

[6] Johnson K., Soto J.T., Glicksberg B., Shameer K., Miotto R., Ali M., Ashley E., Dudley J.T. (2018). Artificial intelligence in cardiology. J. Am. Coll. Cardiol..

[7] Murdoch T.B., Detsky A.S. (2013). The inevitable application of big data to health care. JAMA.

[8] (2013). Administration UFaD. Guidance for Industry: Electronic Source Data in Clinical Investigations. https://www.fda.gov/downloads/drugs/guidances/ucm328691.pdf.

[9] He J., Baxter S.L., Xu J., Xu J., Zhou X., Zhang K. (2019). The practical implementation of artificial intelligence technologies in medicine. Nat. Med..

[10] Tang A., Tam R., Cadrin-Chênevert A., Guest W., Chong J., Barfett J., Chepelev L., Cairns R., Mitchell J.R., Cicero M.D. (2018). Canadian Association of Radiologists White Paper on Artificial Intelligence in Radiology. Can. Assoc. Radiol. J..

[11] Kermany D.S., Goldbaum M., Cai W., Valentim C.C.S., Liang H., Baxter S.L., McKeown A., Yang G., Wu X., Yan F. (2018). Identifying Medical Diagnoses and Treatable Diseases by Image-Based Deep Learning. Cell.

[12] Coppola F., Giannini V., Gabelloni M., Panic J., Defeudis A., Monaco S.L., Cattabriga A., Cocozza M., Pastore L., Polici M. (2021). Radiomics and Magnetic Resonance Imaging of Rectal Cancer: From Engineering to Clinical Practice. Diagnostics.

[13] Scapicchio C., Gabelloni M., Barucci A., Cioni D., Saba L., Neri E. (2021). A deep look into radiomics. Radiol. Med..

[14] Coppola F., Faggioni L., Gabelloni M., De Vietro F., Mendola V., Cattabriga A., Cocozza M.A., Vara G., Piccinino A., Monaco S.L. (2021). Human, All Too Human? An All-Around Appraisal of the “Artificial Intelligence Revolution” in Medical Imaging. Front. Psychol..

[15] Grace K., Salvatier J., Dafoe A., Zhang B., Evans O. (2018). When will AI exceed human performance Evidence from AI experts. J. Artif. Intell. Res..

[16] LeCun Y., Bengio Y., Hinton G. (2015). Deep learning. Nature.

[17] Huang G., Liu Z., Van Der Maaten L., Weinberger K.Q. Densely connected convolutional networks. Proceedings of the IEEE Conference on Computer Vision and Pattern Recognition.

[18] Mura Dataset. https://stanfordmlgroup.github.io/competitions/mura/.

[19] Rajpurkar P., Irvin J., Bagul A., Ding D., Duan T., Mehta H., Yang B., Zhu K., Laird D., Ball R.L. (2017). Mura: Large dataset for abnormality detection in musculoskeletal radiographs. arXiv.

[20] Chada G. (2019). Machine learning models for abnormality detection in musculoskeletal radiographs. Reports.

[21] Varma M., Lu M., Gardner R., Dunnmon J., Khandwala N., Rajpurkar P., Long J., Beaulieu C., Shpanskaya K., Fei-Fei L. (2019). Automated abnormality detection in lower extremity radiographs using deep learning. Nat. Mach. Intell..

[22] Mondol T.C., Iqbal H., Hashem M. (2019). Deep CNN-based ensemble CADx model for musculoskeletal abnormality detection from radiographs. Proceedings of the 2019 5th International Conference on Advances in Electrical Engineering.

[23] Teeyapan K. Abnormality Detection in Musculoskeletal Radiographs using EfficientNets. Proceedings of the of the 24th International Computer Science and Engineering Conference.

[24] El-Saadawy H., Tantawi M., Shedeed H.A., Tolba M.F. (2021). A Hybrid Two-Stage GNG–Modified VGG Method for Bone X-rays Classification and Abnormality Detection. IEEE Access.

[25] He M., Wang X., Zhao Y. (2021). A calibrated deep learning ensemble for abnormality detection in musculoskeletal radiographs. Sci. Rep..

[26] Aziz A.Z.B., Hasan M., Mehedi A., Shin J. (2021). Deep Transfer Learning-Based Musculoskeletal Abnormality Detection 2021. Proceedings of International Joint Conference on Advances in Computational Intelligence.

[27] Kumar Y., Kaur K., Singh G. Machine learning aspects and its applications towards different research areas. Proceedings of the 2020 International Conference on Computation, Automation and Knowledge Management (ICCAKM).

[28] Singh G., Anand D. (2021). CompDNet-512: Hybrid Deep Learning Architecture for Prediction of COVID-19. Proceedings of the 2021 3rd International Conference on Advances in Computing, Communication Control and Networking (ICAC3N).

[29] Salgotra R., Singh S., Singh U., Saha S., Gandomi A.H. (2020). COVID-19: Time series datasets India versus world. Mendeley Data.

[30] Python. https://www.python.org/downloads/release/python-369/.

[31] Ferroukhi M., Ouahabi A., Attari M., Habchi Y., Taleb-Ahmed A. (2019). Medical video coding based on 2nd-generation wavelets: Performance evaluation. Electronics.

[32] Gurpreet S., Vinay C. (2013). Design and Implementation of Testing Tool for Code Smell Rectification Using C-Mean Algorithm. Int. J. Adv. Res. Comput. Sci..

[33] Kingma D.P., Ba J. (2014). Adam: A method for stochastic optimization. arXiv.

[34] Baldi P., Sadowski P.J. Understanding dropout. Proceedings of the 26th International Conference on Neural Information Processing Systems.

[35] Shivangi S., Darpan A. (2017). A novel digital signature algorithm based on biometric hash. Int. J. Comput. Netw. Inf. Secur..

[36] Anand D., Khemchandani V. (2020). Data Security and Privacy Functions in Fog Computing for Healthcare 4.0. Fog Data Analytics for IoT Applications.

[37] Kaur S.S., Darpan A. Smart Health Monitoring During Pandemic using Internet of Things. Proceedings of the 2021 10th IEEE International Conference on Communication Systems and Network Technologies (CSNT).

[38] Kaur S.S., Darpan A. (2021). Emotion Classification and Facial Key point detection using AI. Proceedings of the 2021 2nd International Conference on Advances in Computing, Communication, Embedded and Secure Systems (ACCESS).

[39] Darpan A., Aashish K. (2022). IoT-Based Automated Healthcare System. Advanced Healthcare Systems: Empowering Physicians with IoT-Enabled Technologies.

